# The Effects of the Binder and Buffering Matrix on InSb-Based Anodes for High-Performance Rechargeable Li-Ion Batteries

**DOI:** 10.3390/nano11123420

**Published:** 2021-12-17

**Authors:** Vo Pham Hoang Huy, Il Tae Kim, Jaehyun Hur

**Affiliations:** Department of Chemical and Biological Engineering, Gachon University, Seongnam 13120, Gyeonggi, Korea; vophamhoanghuy@yahoo.com.vn

**Keywords:** InSb, InSb–C, PAA binder, anodes, Li-ion batteries

## Abstract

C-decorated intermetallic InSb (InSb–C) was developed as a novel high-performance anode material for lithium-ion batteries (LIBs). InSb nanoparticles synthesized via a mechanochemical reaction were characterized using X-ray diffraction (XRD), high-resolution transmission electron microscopy (HRTEM), scanning electron microscopy (SEM), X-ray photoelectron spectroscopy (XPS), and energy-dispersive X-ray spectroscopy (EDX). The effects of the binder and buffering matrix on the active InSb were investigated. Poly(acrylic acid) (PAA) was found to significantly improve the cycling stability owing to its strong hydrogen bonding. The addition of amorphous C to InSb further enhanced mechanical stability and electronic conductivity. As a result, InSb–C demonstrated good electrochemical Li-ion storage performance: a high reversible specific capacity (878 mAh·g^−1^ at 100 mA·g^−1^ after 140 cycles) and good rate capability (capacity retention of 98% at 10 A·g^−1^ as compared to 0.1 A·g^−1^). The effects of PAA and C were comprehensively studied using cyclic voltammetry, differential capacity plots, ex-situ SEM, and electrochemical impedance spectroscopy (EIS). In addition, the electrochemical reaction mechanism of InSb was revealed using ex-situ XRD. InSb–C exhibited a better performance than many recently reported Sb-based electrodes; thus, it can be considered as a potential anode material in LIBs.

## 1. Introduction

Lithium-ion batteries (LIBs) have been widely used in various portable devices and energy storage systems owing to their high energy density, high cell voltage, low self-discharge, and low memory effect [1,2,3,4]. Despite these beneficial features, current graphite anodes cannot satisfy the rapidly growing demands for their use in various applications, such as mobile devices, electrical vehicles, and large-scale grid storage systems. Therefore, the development of new anode materials with a high specific capacity, good rate capability, and long service life that can replace low theoretical capacity (372 mAh·g^−1^) commercial graphitic anodes is required [4,5,6,7,8,9,10,11,12,13,14,15,16,17]. Li alloys with elements, such as Si, P, Sn, and Sb, are considered to be promising anode materials because of their higher theoretical capacities (Si: 4200, P: 2595, Sn: 993, and Sb: 660 mAh·g^−1^). However, it is not straightforward to control the large volume change in these materials due to expansion/contraction during lithiation/delithiation, which leads to a deteriorated cell performance [18,19,20,21,22,23].

Recently, Sb-based materials have gained significant attention as promising anodes in LIBs owing to their low cost, high conductivity, high density, and high theoretical capacity [24,25,26,27]. Sb has higher conductivity and stability than P (the same element family) and Si (the material with the highest theoretical capacity), making it a suitable material for the development of high-performance anodes for LIBs. Because of these attractive characteristics, Sb has been intensively investigated for use in LIBs. However, satisfactory performance cannot be achieved using Sb alone because of its high-volume expansion (135%) during the alloying reaction (3Li^+^ + Sb → Li_3_Sb) [28]. Many strategies have been proposed to resolve this problem. 

The formation of a nanoscale Sb-based intermetallic alloy is an effective approach that can improve the cycling stability of Sb-based electrodes. Nanoscale active materials reduce the Li-ion diffusion pathway and alleviate the stress and strain during the electrochemical reaction. In addition, the stepwise electrochemical reaction in bimetallic Sb-based alloy nanoparticles can mitigate a large volume change relative to a pure Sb electrode. Along this line, He et al. demonstrated monodisperse colloidal SnSb nanocrystals (approximately 20 nm) with a discharge capacity of 700 mAh·g^−1^ at 0.5 C after 100 cycles [29]. Yi et al. synthesized morphology-controllable Sn–Sb composites with micro- and nano-sized hollow, dendritic, or mixed-type structures; these designed composites also exhibited good cycling stability and rate performance in LIBs and sodium-ion batteries (SIBs) [29].

Another effective approach that can enhance the performance of Sb-based electrodes is to introduce various nanoscale conductive carbon materials to create nanostructured Sb/C composites (e.g., 1D carbon nanotubes, nanofibers, nanorods, 2D graphene, 3D graphite, and porous carbon) [30,31,32,33]. In this composite, carbon prevents the agglomeration of nanoparticles, increases the electrical conductivity, and reduces the volume change of the active Sb [34,35,36]. Therefore, the cycling stability is notably improved by adding carbon.

The binder is the crucial adhesive between the active material and conductive carbon on the current collector. The adhesion between the active component and the binder is very important during the electrochemical reaction because the stress on the active material caused by volume expansion can weaken the binding force. In a pioneering study on binder materials, Kim et al. studied the effect of a new binder material (a blend of poly(acrylic acid) (PAA) and poly(amide imide) (PAI)) on electrode adhesion and recovery characteristics. They demonstrated that the composite polymer binder exhibited superior properties compared to the individual polymers [37]. Similarly, Choi et al. developed a new polyrotaxane-based binder for active micro-silicon particle batteries in which they achieved a remarkably stable capacity of over 3000 mAh·g^−1^ after 150 cycles [38]. Wu et al. have shown that conductive binders based on polyfluorene (PF) exhibit superior performance owing to their electronic conductivity and mechanical strength [39]. Among the various binders studied, PAA has shown exceptionally good performance for the Si electrode because of (i) its abundant carboxylic acid functional groups (−COOH) that enable strong bonding to the native hydroxyl species on the Si particle surface [40,41]; (ii) good mechanical strength associated with low swelling in a liquid electrolyte [42]; and (iii) the formation of an artificial solid electrolyte interface (SEI) on the Si surface that stabilizes the electrode–electrolyte interface [43]. Accordingly, PAA is expected to be a promising binder for various anode materials with a large volume change in LIBs.

In this study, we demonstrate C-decorated InSb (InSb–C) as a novel Sb-based bimetallic high-performance anode for LIBs. InSb has been widely studied for use in transistors, magnetic sensors, and infrared photodetectors because of its semiconducting properties, which include a narrow band gap (0.17 eV), high electron mobility, and a high density of conduction states [44,45,46]. Although some In-based nanomaterials have been reported as good anode materials owing to the high theoretical capacity of In (1012 mAh·g^−1^) [27,28], intermetallic InSb has rarely been investigated as an anode material for LIBs. To achieve a high-performance InSb electrode, we investigate the effects of the binder and buffering matrix on the performance of InSb. This study demonstrates that PAA is an effective binder that impedes volume expansion and limits the structural degradation of the electrode owing to its strong hydrogen bonding with the active InSb. The addition of amorphous C reduces the stresses in InSb during lithiation/delithiation and increases the electrical conductivity. Therefore, with an appropriate binder and matrix, InSb–C exhibits high performance in terms of specific capacity, cyclic stability, and rate performance. Various characterization techniques are used to elucidate the mechanism behind the improvement, including X-ray diffraction (XRD), scanning electron microscopy (SEM), high-resolution transmission electron microscopy (HRTEM), energy-dispersive X-ray spectroscopy (EDX), Fourier-transform infrared spectroscopy (FTIR), X-ray photoelectron spectroscopy (XPS), and electrochemical impedance spectroscopy (EIS). Furthermore, the phase transformation mechanism of InSb during lithiation/delithiation is studied using ex-situ XRD.

## 2. Experimental Section

### 2.1. Synthesis of InSb and InSb–C

InSb was synthesized using high-energy mechanical milling (HEMM). In (99.99%, Sigma-Aldrich, St. Louis, MO, USA) and Sb (99.998%, Sigma-Aldrich, St. Louis, MO, USA) powders were mixed in a 1:1 molar ratio and then placed in an 80 cm^3^ ZrO_2_ bowl with hardened ZrO_2_ balls in a 20:1 ball-to-powder ratio. The mixture was milled in an Ar atmosphere for 10 h at 300 rpm. InSb–C nanocomposites were prepared using HEMM, where a mixture of as-synthesized InSb and acetylene black powder (99.9%, 100% compressed, specific surface area of 75 m^2^·g^−1^, bulk density of 170–230 g·L^−1^, Alfa Aesar, Catalog No. 045527, Ward Hill, MA, USA) at a mass ratio of 9:1 was milled under the same conditions as the InSb synthesis. The mechanochemical synthesis reaction for InSb–C is described as follows:

In + Sb → InSb + C → InSb–C(1)

### 2.2. Material Characterization

The crystal structures of the as-prepared InSb and InSb–C were measured using powder XRD (D/MAX–2200 Rigaku, Tokyo, Japan) with Cu Kα (λ = 1.54 Å) radiation. The microscopic morphology of the as-synthesized powder materials was observed using HRTEM (JEOL JEM-2100F) and SEM (Hitachi S4700, Tokyo, Japan). XPS (Kratos Axis Anova, Manchester, UK) was used to evaluate the chemical states of the synthesized materials. The elemental content and distribution of the as-prepared powder and electrode after electrochemical reactions were evaluated using EDX.

### 2.3. Electrochemical Measurements

All electrodes were prepared by casting a slurry containing 70% active material, 15% carbon (Super-P, 99.9%, Alfa Aesar), and 15% PAA (Mw 450000, Sigma Aldrich, St. Louis, MO, USA) or a poly(vinylidene fluoride) (PVDF, MW 534000, Sigma Aldrich, St. Louis, MO, USA) binder dissolved in N-Methyl-2-pyrrolidone. The cast electrodes were dried overnight in a vacuum oven at 70 °C and then transferred to an Ar glove box for cell assembly. A coin-type cell (CR2032) was used for half-cell testing. Li metal foil and polyethylene were used as the counter electrode and separating membrane, respectively. The electrolyte was 1 M LiPF_6_ in ethylene carbonate/diethyl carbonate (EC/DEC, 1:1 *v*/*v*). The electrochemical performance of InSb and InSb–C was evaluated using a battery testing system (WBCS3000, WonATech, Seoul, South Korea). The galvanostatic charge–discharge (GCD) profile was measured from 0.01 to 2.5 V (vs. Li/Li^+^). Cyclic voltammetry (CV) at a scanning rate of 0.1 mV·s^−1^ was used to characterize the electrochemical reactions of InSb with Li^+^. The rate capability was measured using a battery cycler (WBCS3000, WonATech, Seoul, South Korea) at current densities of 0.1, 0.5, 1, 3, 5, and 10 A·g^−1^. EIS (ZIVE MP1, WonATech) was measured in the frequency range from 100 kHz to 100 mHz with an AC amplitude of 10 mV.

## 3. Results and Discussion

**Figure 1:**
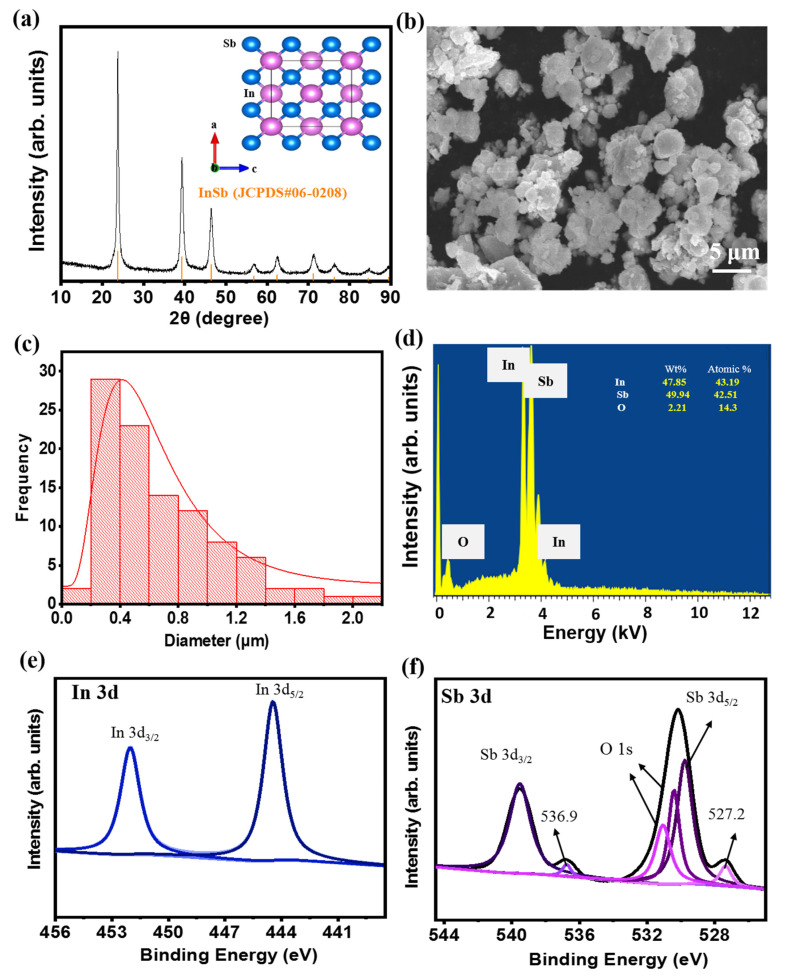
(**a**) XRD pattern (inset: crystalline structure), (**b**) SEM image, (**c**) particle size distribution, and (**d**) EDX spectrum of the as-synthesized InSb powder. XPS profiles of (**e**) In 3d, and (**f**) Sb 3d for the InSb powder.

Figure 1a shows the XRD pattern of the as-prepared InSb powder obtained using the HEMM process. The XRD pattern coincided with the standard data of zinc blende InSb (JCPDS #06-0208) with no detected impurity phases. This indicated that a single phase of the zinc blende structure was successfully obtained, with a lattice constant of 0.646 Å and a space group of T_d_^2^-F43 m, as shown in the inset of Figure 1a. The average crystalline domain size of the as-prepared InSb was calculated to be 0.225 nm using the Scherrer formula (Table S1). The particle size of InSb ranged from hundreds of nanometers to a few micrometers (Figure 1b,c). One of the most important factors affecting the cell performance and safety of LIBs as well as reducing cell aging is the particle size of the active material. The particle size of the material affects the electrochemical performance of the battery [47,48,49]. In general, the small particles have short diffusion pathways (fast Li-ion diffusion), large surface area, and lower overpotential, thus allowing faster C-rate operation and high capacity. However, the beneficial effect of particle size reduction on cell performance is limited to certain particle sizes. The excessively large surface area can lead to large proportion of passivation layers, such as SEI, leading to an irreversible capacity loss [50,51,52]. Considering this, commercial batteries usually contain micrometer-sized particles for the electrode materials. However, the appropriate size of electrode material highly depends on the intrinsic properties of the electrode materials because they have different atomic structures that influence the electrochemical kinetics, Li-ion intercalation capacities, and structural stability. The size of InSb particles (mostly 200–400 nm in Figure 1c) is thought to be effective in terms of Li-ion diffusion kinetics and capacity while restraining the excessive surface passivation (e.g., SEI). EDX analysis of the InSb powder revealed that the elemental ratio of In and Sb was approximately 1:1 (Figure 1d). The presence of O in the InSb powder is due to the partially oxidized surface of the InSb particles. The composition and chemical state of InSb were examined using XPS (Figure 1e,f). The XPS signals observed at 452.1 and 444.5 eV (Figure 1e) can be ascribed to In 3d_3/2_ and In 3d_5/2_, respectively, while the peaks at 539.5 and 530.1 eV (Figure 1f) were indexed to Sb 3d_3/2_ and Sb 3d_5/2_, respectively, verifying the InSb alloy structure after the HEMM process. Meanwhile, the two small peaks at 536.9 and 527.2 eV (Figure 1f) are related to the surface oxidation of the InSb materials, consistent with the EDX analysis results (Figure 1d). The FTIR analysis of the InSb also confirmed the presence of hydroxide functional groups, as shown in Figure S1. The presence of hydroxyl groups on InSb should result in a high affinity for binders with polar functional groups (such as PAA), which can form strong hydrogen bonds. The binder can then serve as an elastic barrier that prevents InSb particles from aggregating while maintaining stable contact between the electrode and current collector during electrochemical reactions.

**Figure 2:**
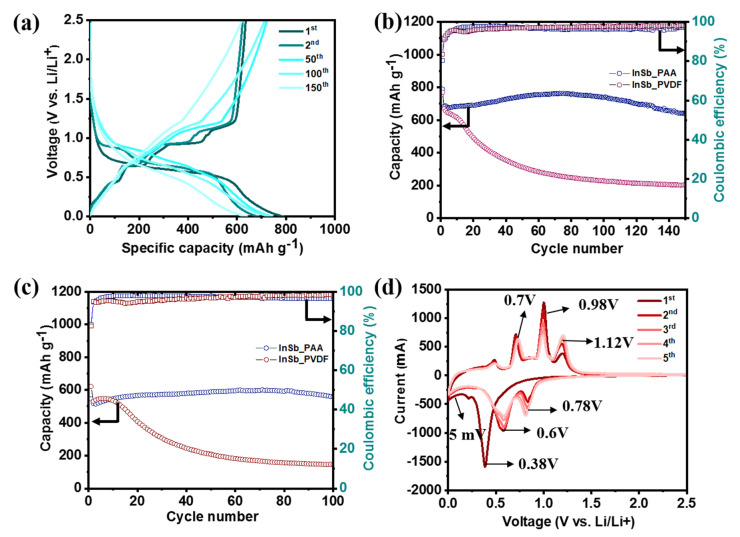
Electrochemical performance of the InSb electrode. (**a**) GCD voltage profiles of InSb_PAA at a current density of 100 mA·g^−1^. Cyclic performance of the InSb_PAA and InSb_PVDF at a current density of (**b**) 100 and (**c**) 500 mA·g^−1^. (**d**) CV curves of InSb_PAA.

The half-cell performance of InSb was measured using two different binders (PAA and PVDF) to investigate its electrochemical behavior (Figure 2). The GCD voltage profiles of InSb_PAA and InSb_PVDF are shown in Figure 2a and Figure S2, respectively. The initial charge/discharge capacities of InSb_PAA and InSb_PVDF were 790/635 and 770/643 mAh·g^−1^, respectively, corresponding to initial coulombic efficiencies (ICEs) of 80.9% and 83.5%. The irreversible capacity losses in the first cycle are associated with the formation of an SEI layer for both electrodes. Although the specific capacities of InSb were not significantly different for PAA and PVDF in the first cycle, a significant capacity reduction was observed for InSb_PVDF during the initial 10 cycles at both low (Figure 2b) and high current densities (Figure 2c). The specific capacities of InSb_PVDF were 203.3 mAh·g^−1^ after 140 cycles and 146.8 mAh·g^−1^ after 100 cycles at 100 and 500 mA·g^−1^, respectively, corresponding to capacity retention values of 30.4% and 27.5%. Moreover, InSb_PAA displayed much better performance in terms of stability and capacity; it exhibited specific capacities of 639.5 mAh·g^−1^ after 140 cycles (93.2% capacity retention) and 558.3 mAh·g^−1^ after 100 cycles (92.3% capacity retention) at 100 and 500 mA·g^−1^, respectively. Figure S3 displays the surface morphologies of pristine InSb_PAA and InSb_PVDF. InSb_PAA showed a more uniform surface with a lower roughness than InSb_PVDF owing to the strong hydrogen-bonding interaction between the hydroxyl groups on the InSb particles and the carboxylate groups in PAA, which is not present in InSb_PVDF. Figure 2d shows the first five CV cycles for InSb_PAA in the voltage range from 0.005 to 3.0 V vs. Li/Li^+^. The initial CV curve was markedly different from those of the subsequent cycles due to the formation of an SEI layer on the electrode surface. In the first discharge step, a significant reduction peak emerged at 0.38 V, indicating the Li intercalation into InSb to form Li_2_Sb and In. The peak emerging at 0.24 V can be due to the reaction between In and Li to form Li_y_In. Thus, after completing the discharge step, Li_2_Sb and Li_2_In appear as final products. In the charge process, three oxidation peaks were observed at voltages of 0.70, 0.98, and 1.12 V. Among them, the first peak at 0.70 V corresponds to the complete exclusion of Li, reverting Li_2_In into In. When the anode was charged to 0.98 and 1.12 V, In began to intrude into Li_2_Sb to form InSb. The detailed analysis of this phase transformation will be discussed in the ex-situ analyses. However, the curves nearly overlapped after the second cycle, demonstrating the high reversibility and stability of InSb_PAA. Compared to InSb_PAA, InSb_PVDF showed relatively unstable CV curves with polarized oxidation and reduction peaks even after the second cycle (Figure S4).

**Figure 3:**
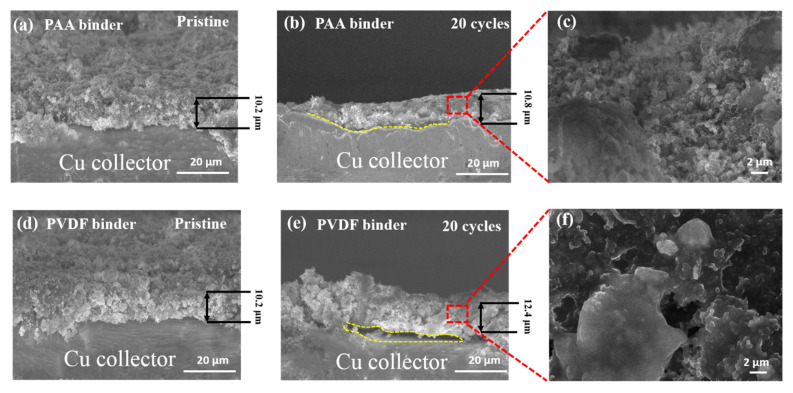
Comparison of the InSb_PAA and InSb_PVDF electrodes before and after 20 cycles. Cross-sectional images of (**a**) pristine InSb_PAA, (**b**,**c**) InSb_PAA after 20 cycles at different magnifications, (**d**) pristine InSb_PVDF, and (**e**,**f**) InSb_PVDF after 20 cycles at different magnifications. The dashed yellow lines in (**b**,**e**) indicate the boundary between electrode and Cu collector.

Figure 3 compares the cross-sectional SEM images of InSb_PAA and InSb_PVDF in the pristine state and after 20 cycles. Although the thicknesses of InSb_PAA and InSb_PVDF were similar in the pristine states (10.2 µm in Figure 3a,d), InSb_PAA was thinner (10.8 µm in Figure 3b) than InSb_PVDF (12.4 µm in Figure 3e) after 20 cycles, indicating a smaller volume expansion of the InSb_PAA. In addition, the InSb_PAA maintained close contact between the electrode and current collector after 20 cycles (Figure 3c). However, the InSb_PVDF electrode partially delaminated from the current collector (Figure 3e) and aggregated (Figure 3f), because it failed to accommodate the large volume change of the InSb particles during repeated electrochemical reactions. These results justify the selection of PAA as an appropriate binder material for the InSb electrode.

**Figure 4:**
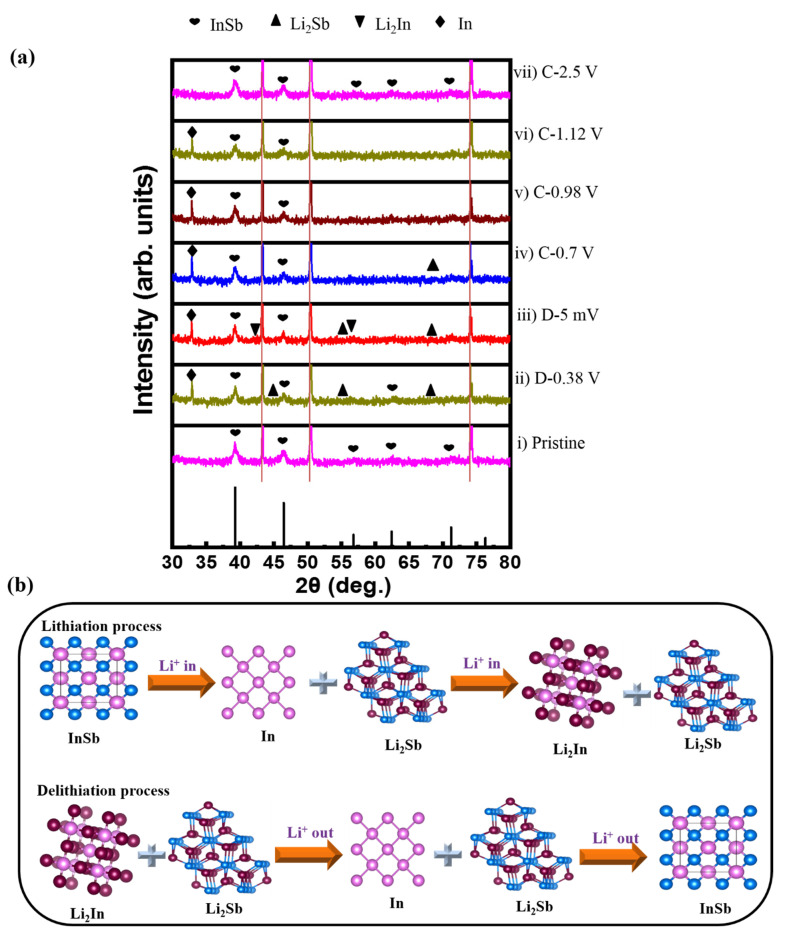
(**a**) XRD patterns collected at selected potential states in the initial lithiation/delithiation process. (**b**) Schematic of the electrochemical reaction mechanism of the InSb_PAA electrode during cycling.

Ex-situ XRD was used to investigate the electrochemical reaction mechanism during the initial lithiation/delithiation process of the InSb electrode (Figure 4a). At a discharge voltage of 0.38 V (D-0.38 V), peaks corresponding to Li_2_Sb and In emerged. When fully discharged (D-5 mV), Li_2_In peaks appeared, while Li_2_Sb and In peaks remained. Upon charging to 0.7 V (C-0.7 V), the Li_2_In phase disappeared. At the charging states of 0.98 and 1.12 V the Li_2_Sb phase vanished. When fully charged to 2.5 V (C-2.5 V), only the peaks matching with InSb re-emerged. The structural transformation of InSb during lithiation/delithiation is summarized as follows: 

Discharging:

InSb → Li_2_Sb + In → Li_2_Sb + Li_2_In + In (partly) (2)

Charging:

Li_2_Sb + Li_2_In + In (partly) → In + Li_2_Sb → InSb (3)

Notably, the InSb phase (major peaks at 39.9° and 46.5°) fully recovered without any impurity peaks after the first cycle, indicating a highly reversible reaction of InSb with Li ions. This likely correlates with the robust binding between InSb and PAA, which effectively protects the active material from pulverization and delamination caused by volume changes. The ex-situ XRD results demonstrate the conversion and alloying/dealloying mechanism of the InSb electrode during discharge/charge, as schematically illustrated in Figure 4b.

Despite the better performance of InSb_PAA compared to that of InSb_PVDF, the InSb_PAA electrode still had a gradual decrease in capacity after ~80 cycles when measured at 100 mA·g^−1^ (Figure 2b). This behavior is also reflected in the coulombic efficiency (CE) variation (Table S2), where the CE steadily increased until ~60 cycles, then decreased afterwards. This might be associated with increasing side reactions between InSb_PAA and the electrolyte as the electrode was cycled. These side reactions can be further explained by a differential capacity plot (DCP) analysis of the initial 140 cycles (Figure S5). From this analysis, the main reduction (at ~0.86 and ~0.92 V) and oxidation (at ~0.59 and 0.81 V) peaks remained unchanged for 80 cycles, but then became broader and shifted after 80 cycles. This polarization leads to inefficient lithiation/delithiation and a progressive capacity drop after 80 cycles. A similar trend was observed at a high current density (Figure 2c). In this case, the capacity gradually increased until 80 cycles, followed by a slight decrease in subsequent cycles. This trend was also observed in the CE variation (Table S3) and DCP analysis (Figures S6 and S7), where the intensities of the oxidation (at ~0.59 and 0.81 V) and reduction (at ~0.86 and ~0.92 V) peaks generally increased for 60 cycles with a negligible polarization (Figure S6), then decreased in intensity after 60 cycles with a slight polarization (Figure S7). Therefore, the electrochemical performance of InSb_PAA at current densities of 100 and 500 mA·g^−1^ was not fully satisfactory, based on these results.

**Figure 5:**
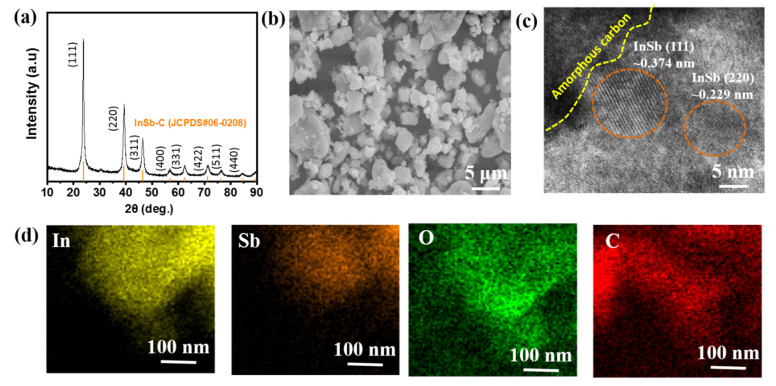
(**a**) XRD pattern, (**b**) SEM image, (**c**) HRTEM image, and (**d**) EDX elemental maps of In, Sb, O, and C of InSb–C.

High-performance LIB anode materials frequently use C decoration to overcome the disadvantages of the active materials. Amorphous C provides improved electrical conductivity and acts as a buffer for withstanding the volume change of Li-active materials [53,54,55,56]. Therefore, InSb–C (or InSb–C_PAA) was prepared by two sequential steps of HEMM (adding acetylene black to the InSb electrode in the secondary HEMM). The XRD peaks of the as-prepared InSb–C matched well with those of InSb (Figure 5a). The size and shape of the InSb–C were almost unchanged compared to those of InSb (Figure 5b). The presence of InSb nanocrystallites was confirmed with HRTEM, with interplanar distances of 0.374 nm and 0.229 nm ((111) and (220) phases of InSb, respectively (Figure 5c)), which was consistent with the XRD analysis. The elemental mapping images (Sb, In, and C) revealed evenly distributed constituent elements (Figure 5d). In addition, the uniform distribution of O confirmed the oxidation of the functional groups on the InSb–C, as in the case of InSb.

**Figure 6:**
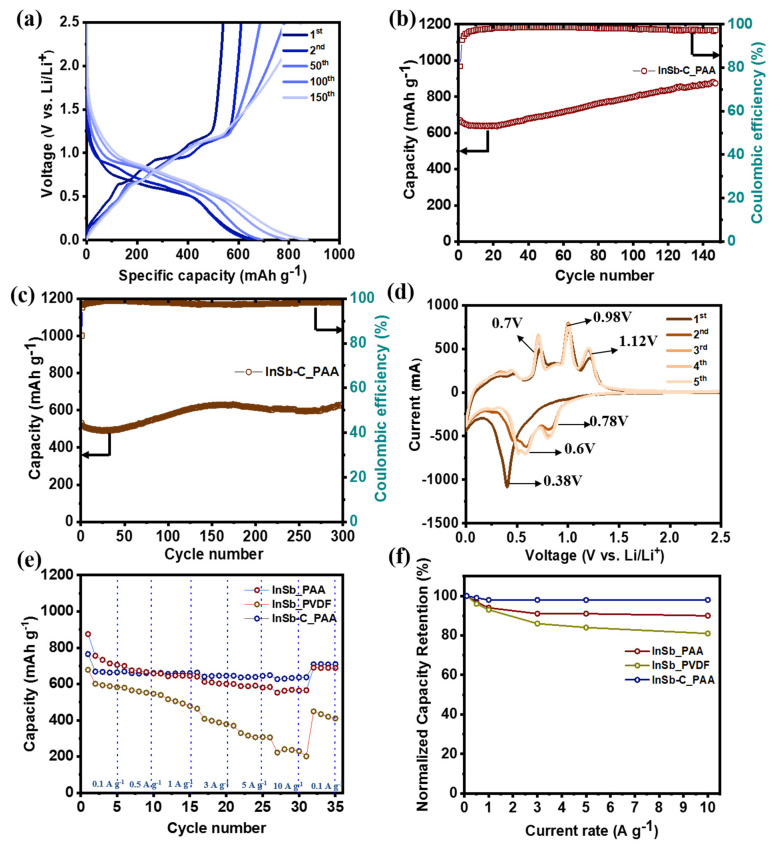
Electrochemical performance of the half-cells. (**a**) GCD profiles of InSb-C_PAA at a current density of 100 mA·g^−1^, cyclic performance of InSb-C_PAA at (**b**) 100 mA·g^−1^ and (**c**) 500 mA·g^−1^, (**d**) CV curves of InSb-C_PAA, (**e**) rate capabilities of the composites, and (**f**) capacity retention of the composites at different current densities.

The electrochemical performance of the InSb–C_PAA electrode is shown in Figure 6. The initial charge/discharge capacity of InSb–C_PAA was 669/540 mAh·g^−1^ with an ICE of 80.7% (Figure 6a). From the EDX analysis (Figure S8) and the calculated theoretical capacity of the individual components (Table S4), the capacity contribution from C in the InSb electrode was estimated to be ~10%. Therefore, the capacity of the electrode was mainly from the active InSb (90% of the total capacity) while C mainly functioned as a buffer matrix (10% capacity contribution) that mitigated the volume expansion of the electrode. Remarkably, the measured capacities of InSb_PAA and InSb–C_PAA were higher than their theoretical capacities (454 and 435.6 mAh·g^−1^, respectively, as calculated in Table S5). This additional capacity is most likely due to electrolyte decomposition and interfacial Li-ion storage. Although the specific capacity of InSb–C_PAA was lower than that of InSb_PAA in the initial cycle, the long-term performance of InSb–C_PAA was superior to that of InSb_PAA. In particular, InSb–C_PAA delivered 878 and 634 mAh·g^−1^ at 100 mA·g^−1^ (Figure 6b) and 500 mAg^−1^ (Figure 6c) after 150 and 300 cycles, respectively. Notably, a steady capacity increase was observed for InSb–C_PAA during the repeated discharge/charge processes, which was attributed to the creation of a polymer-gelled film from electrolyte decomposition and interfacial Li-ion storage [57,58,59]. Furthermore, the variations in the DCP profiles as a function of cycle number were studied at current densities of 100 and 500 mA·g^−1^ to better understand the steady rise in capacity (Figure S9). In the DCP curves of the InSb–C_PAA electrodes, the overall intensity of the redox potentials increased with increasing cycle number. There was also a minor positive shift in the reduction peaks (at 0.86 and 0.92 V) and a slight negative shift in the oxidation peaks (at 0.59 and 0.81 V) in the capacity-increasing region. The degree of polarization in InSb–C_PAA was much lower than that of InSb–PAA (Figure S9). Figure S10 compares the CE variation in InSb–C_PAA and InSb_PAA at current densities of 100 and 500 mA·g^−1^. The detailed CE values are summarized in Table S6 (at 100 mA·g^−1^) and Table S7 (at 500 mA·g^−1^) for the InSb_PAA, InSb_PVDF, and InSb–C_PAA electrodes during the first 10 cycles. As seen in Table S6, InSb–C_PAA had a slightly lower ICE (80.58%) than the InSb_PAA (ICE = 81.42%) and InSb_PVDF electrodes (ICE = 83.53%). However, the CE of the InSb–C_PAA electrode significantly increased after the first cycle, exhibiting the highest CE among the three different electrodes. This trend was also observed at high current densities (Table S7). The high CE of the InSb–C_PAA electrode after the first cycle indicated a high reversibility of lithiation/delithiation. Figure 6d shows the first five CV curves of InSb–C_PAA. In contrast to InSb_PAA and InSb_PVDF, the CV curves of InSb–C_PAA nearly overlapped after the second cycle, exhibiting exceptional cycling stability. The redox peak positions were exactly identical to those observed for InSb_PAA (Figure 2d), indicating that InSb is the main active material. The rate performance (Figure 6e) and normalized capacity retention (Figure 6f) of InSb–C_PAA were measured at various current densities. The average discharge capacities of InSb–C_PAA were 669, 660, 659, 645, 644, and 635 mAh·g^−1^ at current densities of 0.1, 0.5, 1.0, 3.0, 5.0, and 10.0 A·g^−1^, respectively (Figure 6e), which were significantly greater than those of InSb_PAA and InSb_PVDF. Remarkably, even at a high current density of 10 A·g^−1^, the capacity retention of InSb–C_PAA was as high as 98% of its initial capacity (Figure 6f). Even at the current densities higher than 10 A·g^−1^, InSb–C_PAA still presented outstanding electrochemical performance with average specific capacities were 627 and 541 mAh·g^−1^ at 15 and 20 A·g^−1^, respectively (Figure S11). In addition, a high-capacity retention (94.4%) was achieved when the discharge rate was returned to 0.1 A·g^−1^ from 10 A·g^−1^, demonstrating the good rate performance of InSb–C_ PAA. 

**Figure 7:**
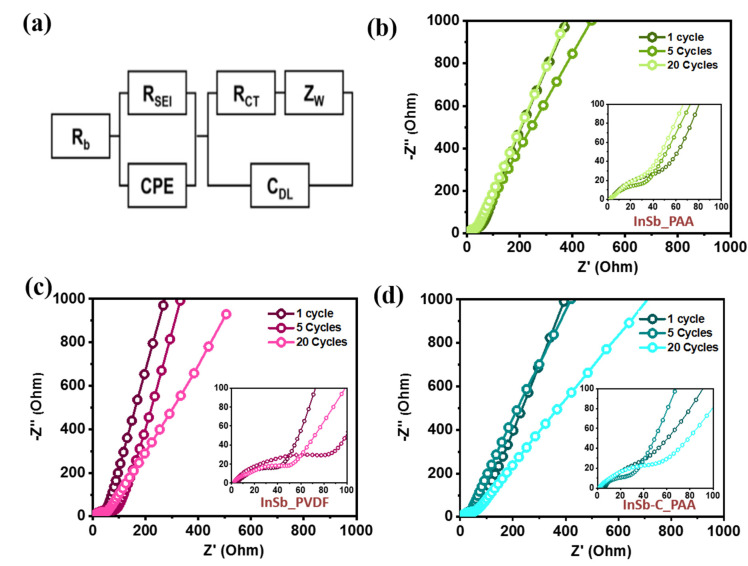
(**a**) The equivalent circuit. Nyquist plots after 1, 5, and 20 cycles for (**b**) InSb_PAA, (**c**) InSb_PVDF, and (**d**) InSb–C_PAA.

EIS profiles of the InSb_PAA, InSb_PVDF, and InSb–C_PAA electrodes were obtained at the 1st, 5th, and 20th cycles (Figure 7). The electrolyte resistance (R_b_), SEI layer resistance (R_SEI_), charge-transfer resistance (R_ct_), interfacial double layer capacitance (C_dl_), constant phase element (C_PE_), and Warburg impedance (Z_w_) are all included in the simplified equivalent circuit depicted in Figure 7a. The compressed semi-circles in the mid-frequency region of the Nyquist plots correspond to R_ct_ at the electrode–electrolyte interface. The cells containing the electrode with the PAA binder (InSb_PAA and InSb–C_PAA) showed decreasing semicircles in the low-frequency region with an increase in the cycle number (from 1 to 20 cycles), indicating a gradual decrease in R_ct_ and steady stabilization of the electrode (Figure 7b,d). The R_ct_ values of InSb_PAA and InSb–C_PAA (Figure 7b,d) were significantly lower than that of InSb_PVDF (Figure 7c). After 20 cycles, InSb–C_PAA exhibited the lowest R_ct_ value among the electrodes (Table S8). These results help to explain the gradual increase in capacity and performance of the InSb–C_PAA electrode during long-term cycling.

**Figure 8:**
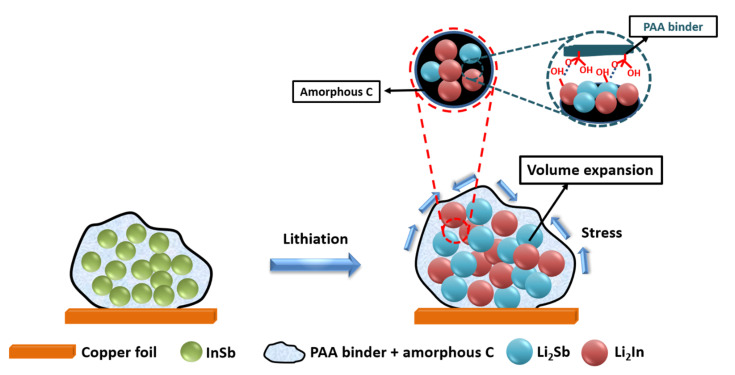
Illustration of the InSb–C_PAA reaction mechanism.

Considering all the results, the Li-ion storage mechanism of the InSb–C_PAA electrode is schematically presented in Figure 8. The overall electrochemical reaction is written as InSb + 4Li^+^ + 4e^−^ ⇄ Li_2_Sb + Li_2_In, neglecting the small capacity contribution from the C matrix. As the discharge proceeds, Li_2_Sb and Li_2_In are formed as products after the reaction with Li ions. During this reaction, a large volume expansion (Li_2_Sb (~135%) and Li_2_In (~297%)) causes mechanical stress on the active InSb. Under prolonged cycles, accumulated stress can result in particle agglomeration, pulverization, and delamination. These issues were effectively resolved by employing a PAA binder and a C buffering matrix. PAA is a binder with numerous COOH functional groups that can form hydrogen bonds with OH groups on the surfaces of active materials (as determined using FTIR (Figure S1) and XPS analyses (Figure 1e)), thereby stabilizing the electrode structure. The presence of amorphous C around InSb facilitates charge transport and provides a mechanical buffer for the active InSb. Therefore, the synergistic effect between the PAA binder and amorphous C contributes to a significant improvement in the electrochemical performance of InSb. Consequently, the performance of the InSb–C_PAA electrode is better than that of most previously reported Sb-based electrodes (Table 1). 

## 4. Conclusions

In summary, InSb and InSb–C were successfully synthesized via HEMM and studied as potential anodes for LIBs. The crystal structure, morphology, and chemical state of these materials were characterized using XRD, SEM, HRTEM, EDX, and XPS. Electrochemical measurements revealed that the PAA binder played a significant role in improving the performance of the InSb-based electrode over conventional PVDF owing to the formation of hydrogen bonds with InSb, which contributed to the strong adhesion between the active materials and current collectors. The addition of amorphous C to InSb improved the mechanical stability and electrical conductivity. As a result, InSb–C_PAA electrodes delivered a high reversible specific capacity (878 mAh·g^−1^ at 100 mA·g^−1^ after 140 cycles) and good rate capability (capacity retention of 98% at 10 A·g^−1^ as compared to 0.1 A·g^−1^), which outperforms most of the Sb-based electrodes recently reported. The synergistic effect of the PAA binder and amorphous C is responsible for the improved electrochemical performance of InSb–C_PAA. Therefore, InSb–C_PAA can be considered as a potential anode material for next-generation LIBs.

## Figures and Tables

**Table 1:** Performance comparison of intermetallic Sb-based anodes for LIBs.

Anode	Cycling Performance	Rate Capability	Synthesis Method	Ref.
Cu_2_Sb	290 mAh·g^−1^ after 25 cycles	-	Ball milling	[60]
Mo_3_Sb_7_	350 mAh·g^−1^ after 100 cycles at 0.12 C	300 mAh·g^−1^ at 100 C	Furnace	[61]
CoSb	448 mAh·g^−1^ after 1000 cycles at 0.66 A·g^−1^	-	Facile colloidal synthesis	[62]
NiSb@C	405 mAh·g^−1^ after 1000 cycles at 0.1 A·g^−1^	393 mAh·g^−1^ at 2.0 A·g^−1^	Freezing drying	[63]
NiSb hollow nanosphere	420 mAh·g^−1^ after 50 cycles at 0.1 A·g^−1^	352 mAh·g^−1^ at 0.8 A·g^−1^	Galvanic replacement reaction	[64]
NiSb/C nanosheet	393 mAh·g^−1^ after 1000 cycles at 2 C	325 mAh·g^−1^ at 10 C	Hydrothermal low-temperature carbothermic reduction	[65]
SnSb@Carbon fiber	674 mAh·g^−1^ after 100 cycles at 0.1 A·g^−1^	163 mAh·g^−1^ at 1.6 A·g^−1^	Electrospinning	[66]
ZnSb/C	481 mAh·g^−1^ after 240 cycles at 0.1 A·g^−1^	426 mAh·g^−1^ at 0.5 A·g^−1^	Annealing	[67]
TiSb_2_	420 mAh·g^−1^ after 120 cycles at 1 C	300 mAh·g^−1^ at 12 C	Furnace	[68]
InSb_PAA InSb–C_PAA	640 mAh·g^−1^ after 140 cycles 846 mAh·g^−1^ after 150 cycles at 0.1 A·g^−1^	594 mAh·g^−1^ at 10 A·g^−1^ 716 mAh·g^−1^ at 10 A·g^−1^	Ball milling	This work

## Data Availability

Not applicable.

## Supplementary Materials

The following are available online at https://www.mdpi.com/article/10.3390/nano11123420/s1, Figure S1: FT-IR results of InSb powder; Figure S2: GCD curves of InSb_PVDF; Figure S3: SEM images of (a and b) InSb_PAA, (c and d) InSb_PVDF binder at different magnification; Figure S4: CV curves of InSb_PVDF from first to fifth cycle; Figure S5: DCP of InSb_PAA during 140 cycles measured at 100 mA·g^−1^: (a) 1−60 cycle, (b) 80−140 cycle. Enlarged view of (c) reduction peak and (d) oxidation peak; Figure S6: (a) DCP of InSb_PAA during initial 60 cycles measured at 500 mA·g^−1^. Enlarged view of (b) oxidation peak and (c) reduction peak; Figure S7: (a) DCP of InSb_PAA from 60 th to 100 th cycle measured at 500 mA·g^−1^. Enlarged view of (b) oxidation peak and (c) reduction peak; Figure S8: EDX spectrum of synthesized InSb–C; Figure S9: DCP profiles of InSb–C_PAA electrodes at current density of (a) 100 mA·g^−1^ during 140 cycles and (b) 500 mA·g^−1^ during 300 cycles; Figure S10: Coulombic efficieny of InSb_PAA, InSb_PVDF, and InSb–C_PAA at current density of (a) 100 and (b) 500 mA·g^−1^; Figure S11: Cyclic performance of InSb-C_PAA at 15 A·g^−1^ and 20 A·g^−1^; Table S1: Crystallite size of InSb calculated using Scherrer equation; Table S2: Coulombic efficiency variation of InSb_PAA at various cycle numbers measured at 100 mA·g^−1^; Table S3: Coulombic efficiency variation of InSb_PAA at various cycle numbers measured at 500 mA·g^−1^; Table S4: Calculation of capacity contribution of InSb and C in the InSb-C composite; Table S5: Calculation of theoretical capacity of InSb and InSb-C; Table S6: Coulombic efficiency of InSb_PAA, InSb_PVDF, and InSb-C_PAA at current density of 100 mA·g^−1^ for initial 10 cycles; Table S7: Coulombic efficiency of InSb_PAA, InSb_PVDF, and InSb–C_PAA at current density of 500 mA·g^−1^ for initial 10 cycles; Table S8: The charge-transfer resistance (R_ct_) of InSb_PAA, InSb_PVDF, InSb-C_PAA.
