# Supplementary material for: The Effects of the Binder and Buffering Matrix on InSb-Based Anodes for High-Performance Rechargeable Li-Ion Batteries
Source: Nanomaterials (Basel). 2021 Dec 17;11(12):3420. doi: 10.3390/nano11123420 (PMC8707395; doi:10.3390/nano11123420)
Supplement: Supplementary file 1 [file nanomaterials-11-03420-s001.zip › nanomaterials-1502963-supplementary.pdf]

Supplementary Information

The Effects of the Binder and Buffering Matrix on InSb-based Anodes for High-Performance Rechargeable Li-Ion Batteries

Vo Pham Hoang Huy, Il Tae Kim * and Jaehyun Hur *

Department of Chemical and Biological Engineering, Gachon University, Seongnam, Gyeonggi 13120, Korea; vophamhoanghuy@yahoo.com.vn

* Correspondence: itkim@gachon.ac.kr (I.T.K.); jhhur@gachon.ac.kr (J.H.); Tel.: +82-31-750-8835 (I.T.K.); +82-31-750-5593 (J.H.)

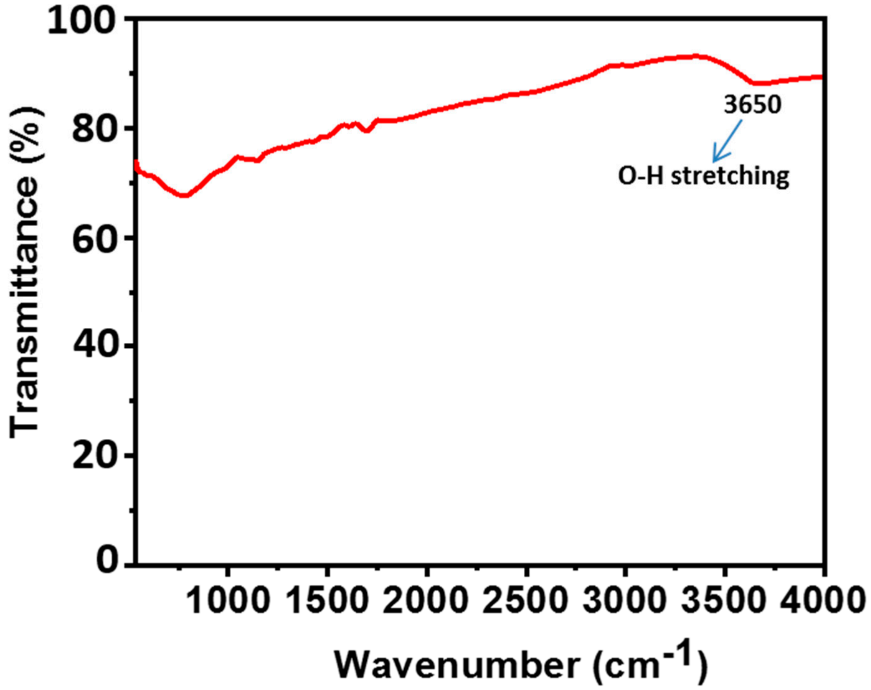

Figure S1. FT-IR results of InSb powder.

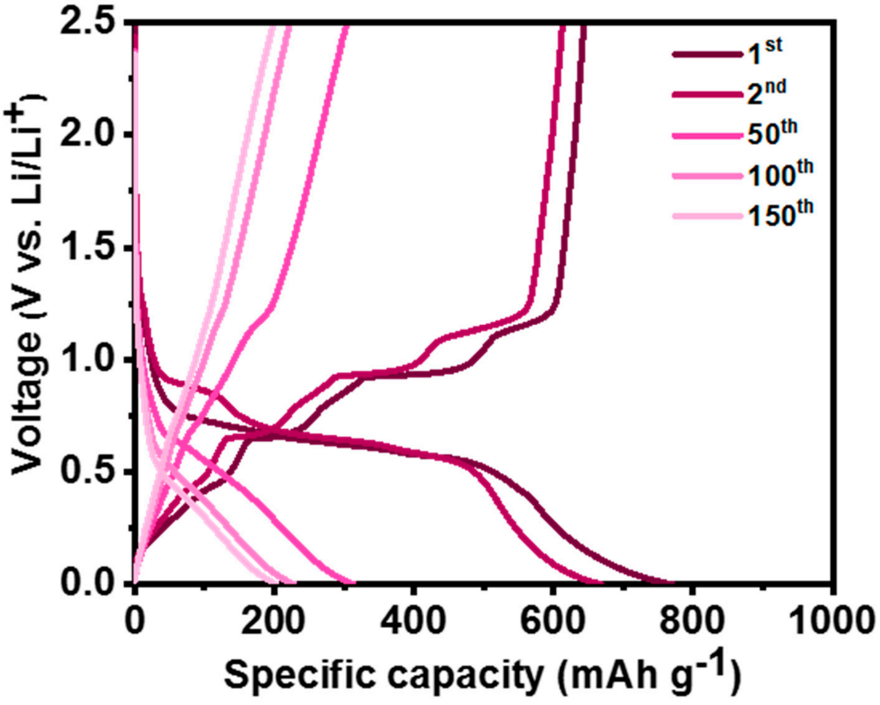

Figure S2. GCD curves of InSb_PVDF.

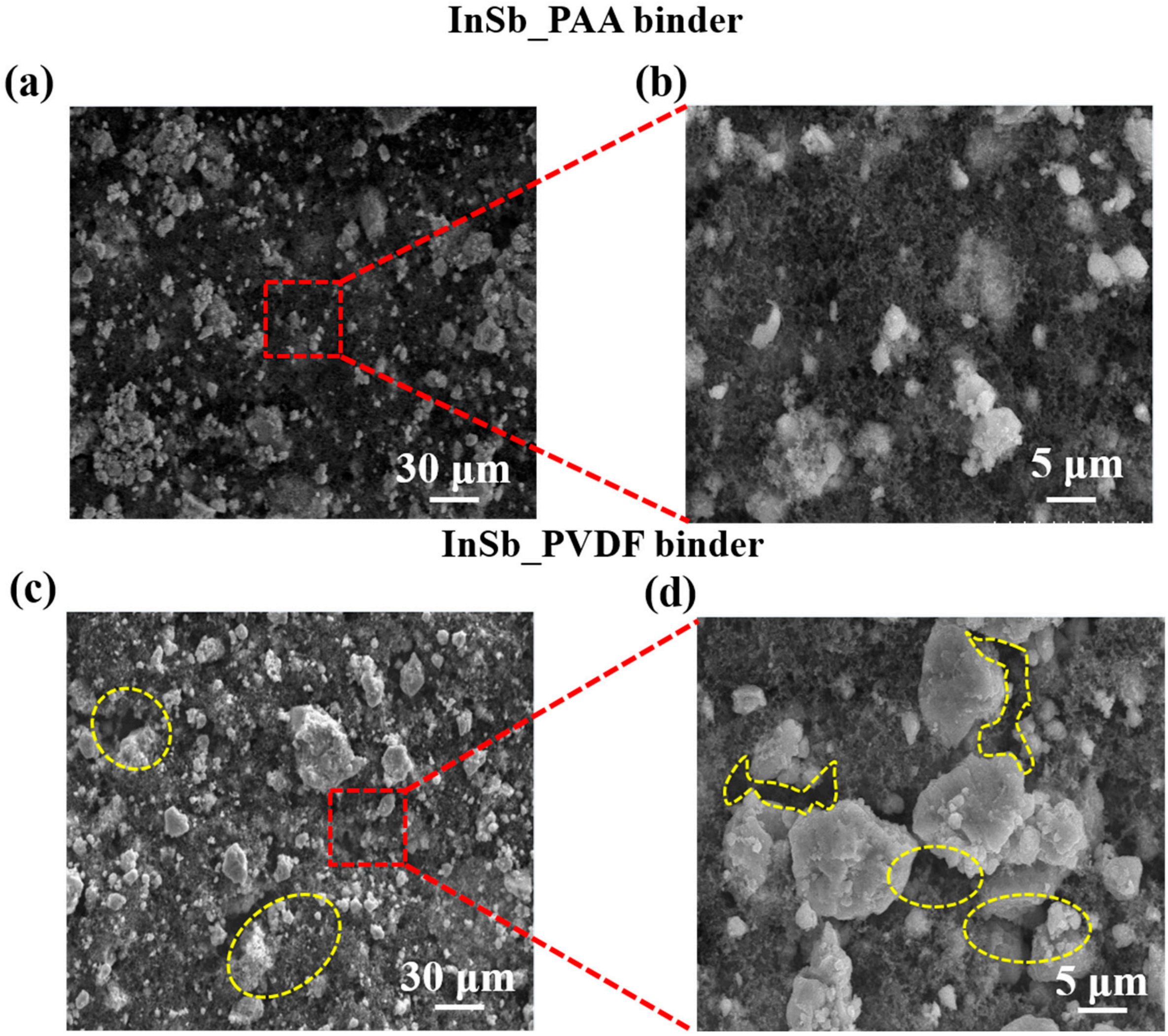

Figure S3. SEM images of (**a**,**b**) InSb_PAA, (**c,d**) InSb_PVDF binder at different magnification.

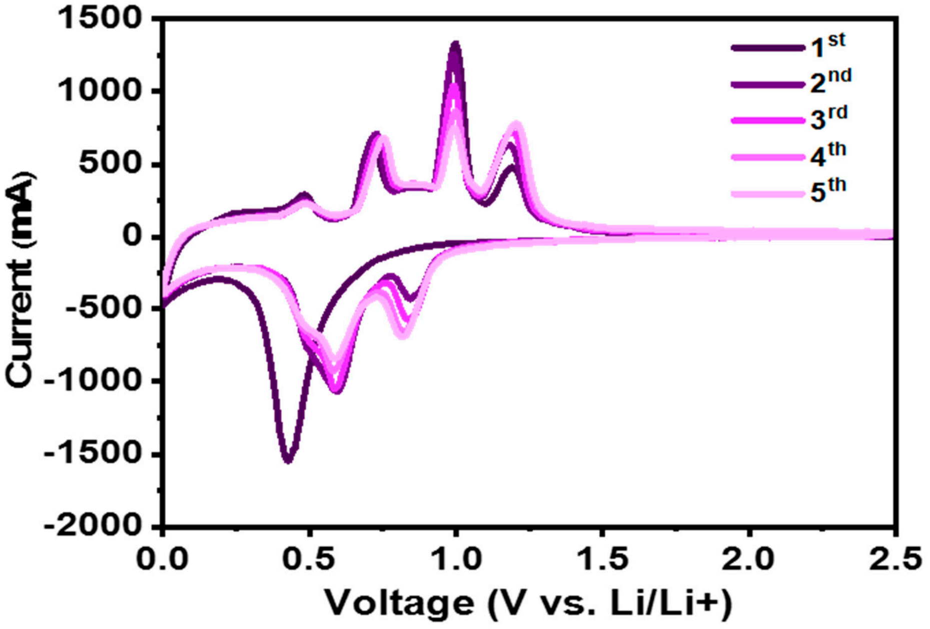

Figure S4. CV curves of InSb_PVDF from first to fifth cycle.

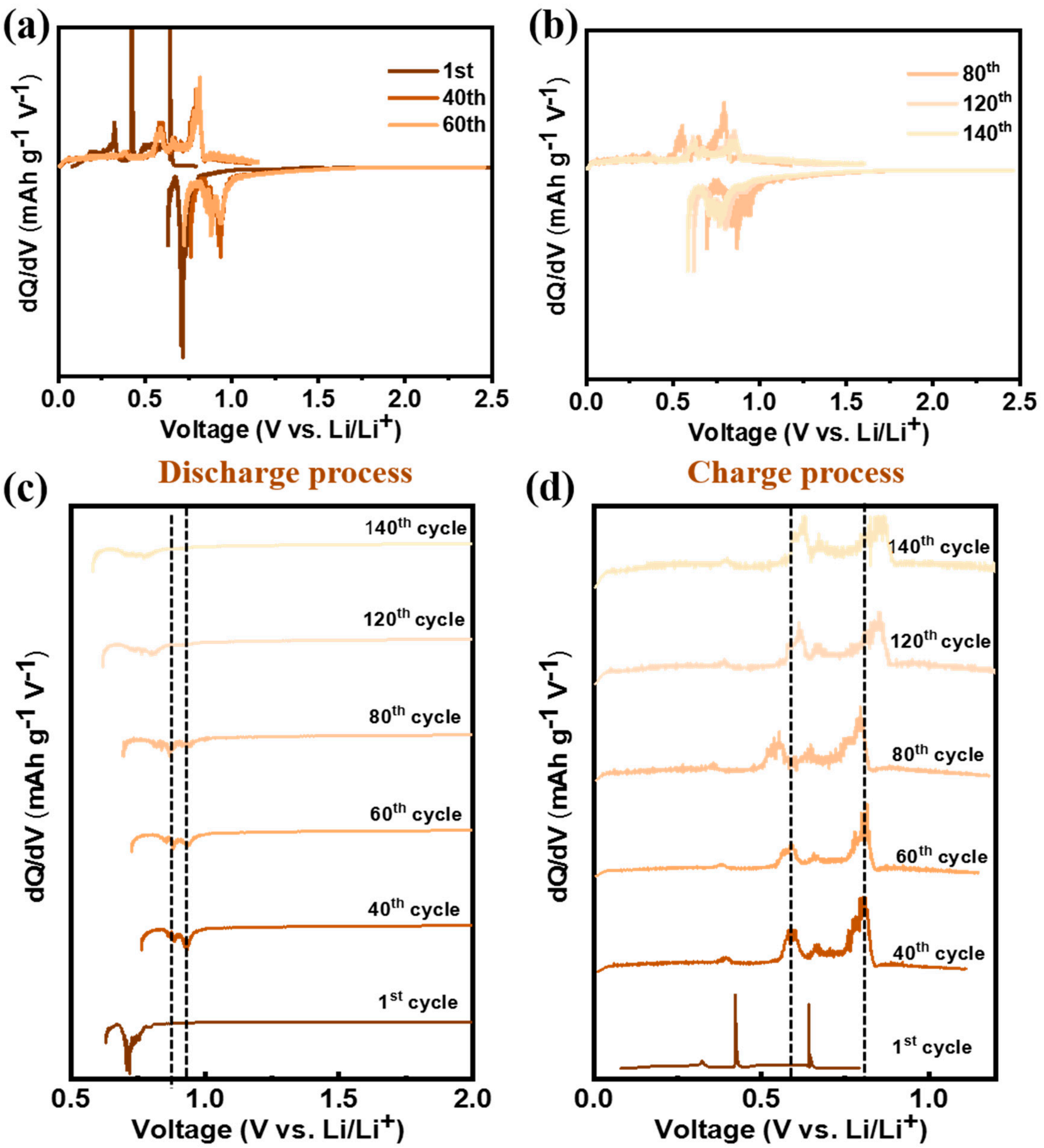

Figure. S5. DCP of InSb_PAA during 140 cycles measured at 100 mA g^{-1}: (**a**) 1−60 cycle, (**b**) 80−140 cycle. Enlarged view of (**c**) reduction peak and (**d**) oxidation peak.

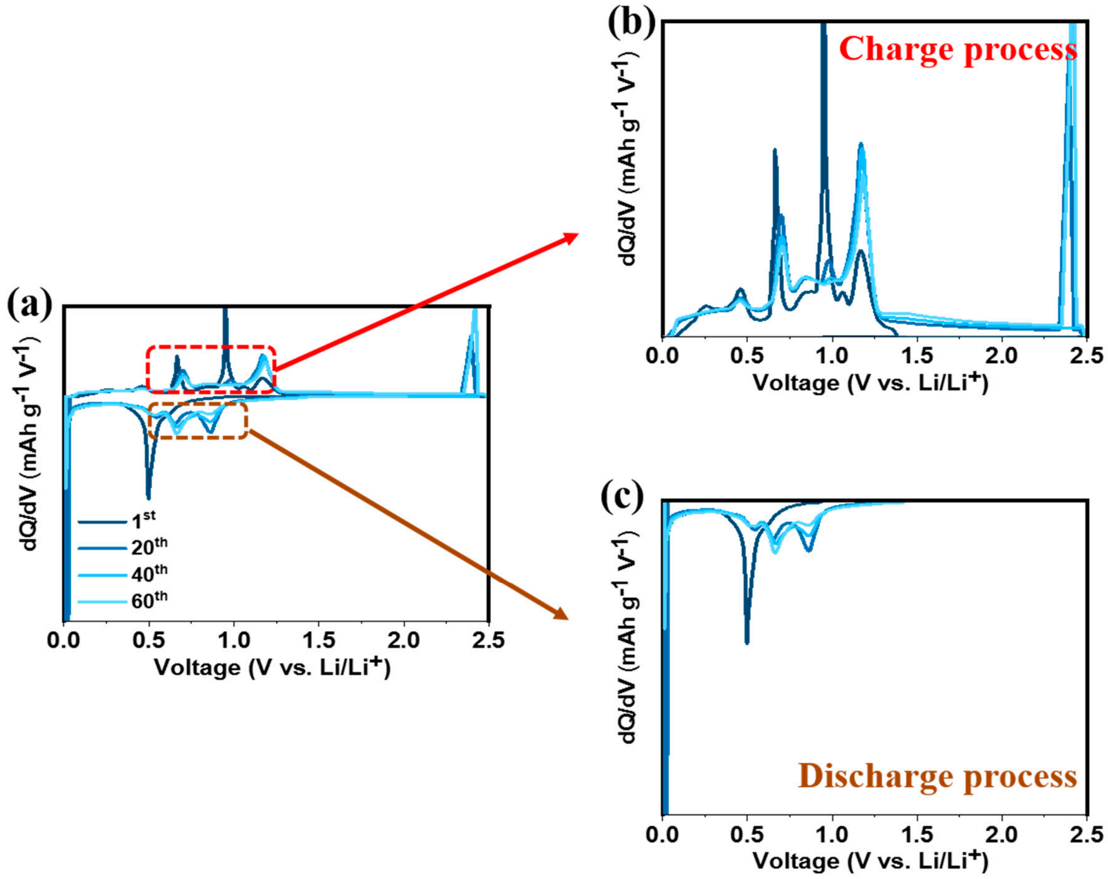

Figure S6. (**a**) DCP of InSb_PAA during initial 60 cycles measured at 500 mA g^{-1}. Enlarged view of (**b**) oxidation peak and (**c**) reduction peak.

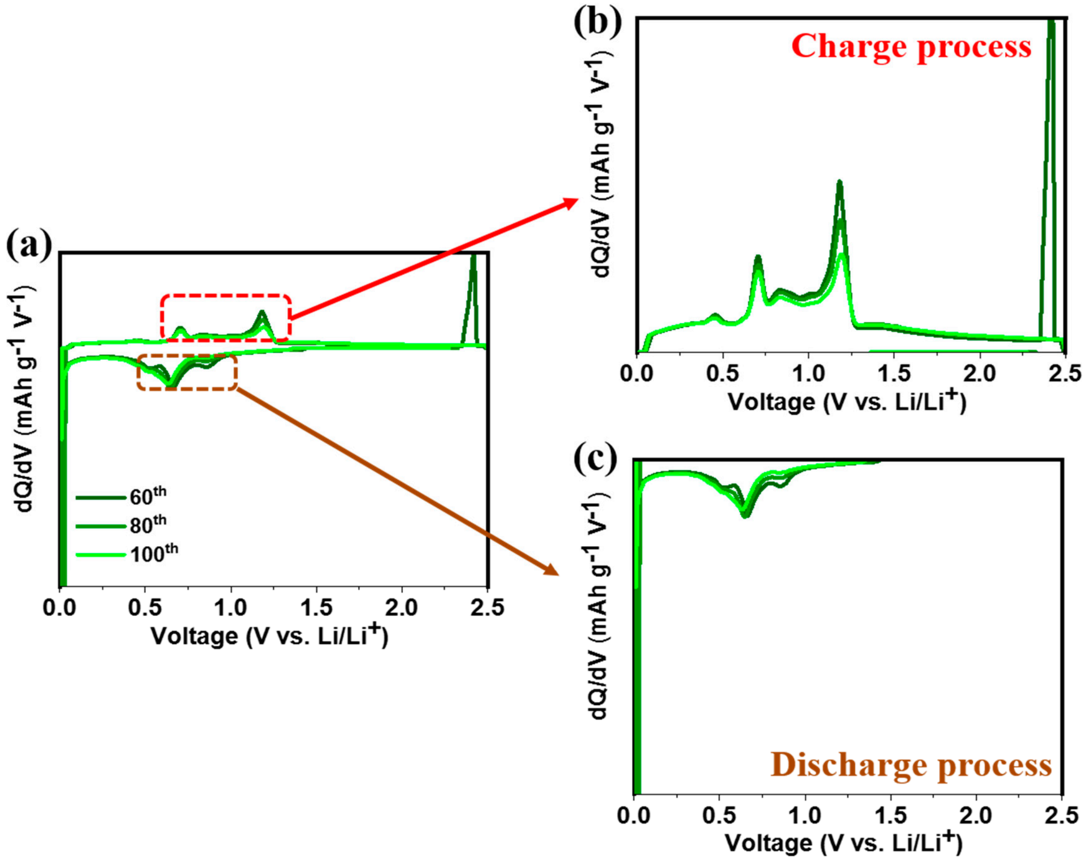

Figure S7. (**a**) DCP of InSb_PAA from 60th to 100th cycle measured at 500 mA g^{-1}. Enlarged view of (**b**) oxidation peak and (**c**) reduction peak.

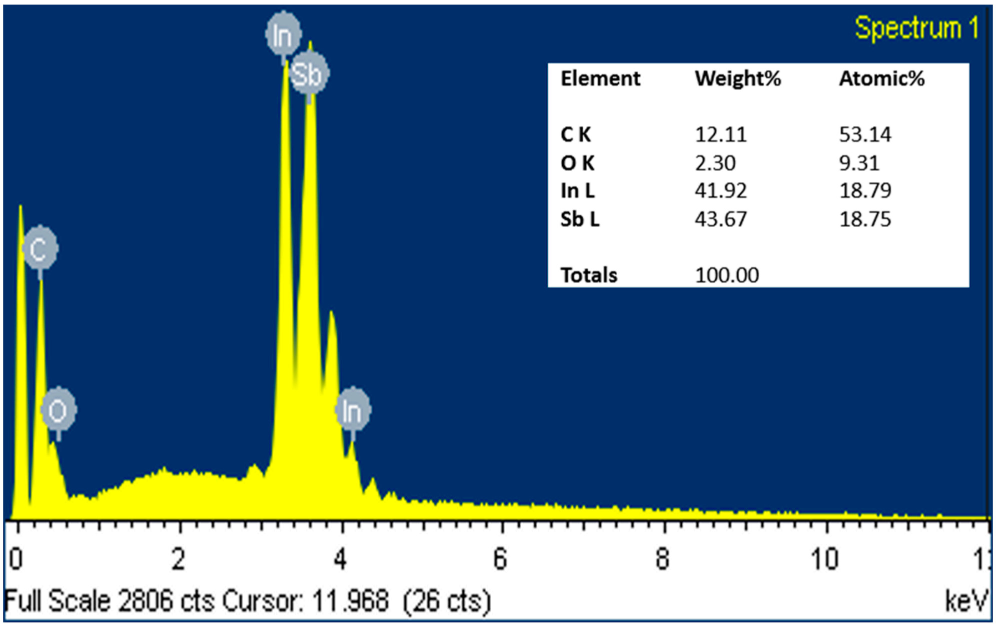

Figure S8. EDX spectrum of synthesized InSb–C.

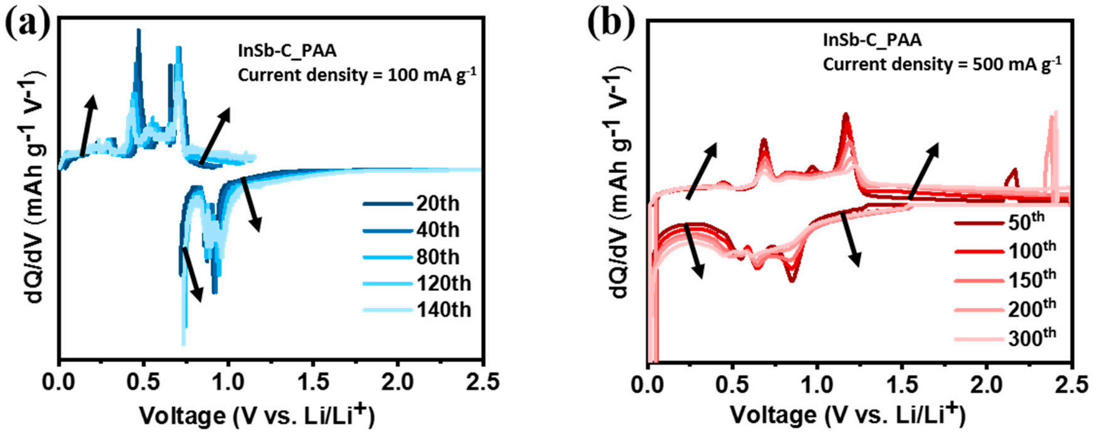

Figure S9. DCP profiles of InSb–C_PAA electrodes at current density of (**a**) 100 mA g^{-1} during 140 cycles and (**b**) 500 mA g^{-1} during 300 cycles.

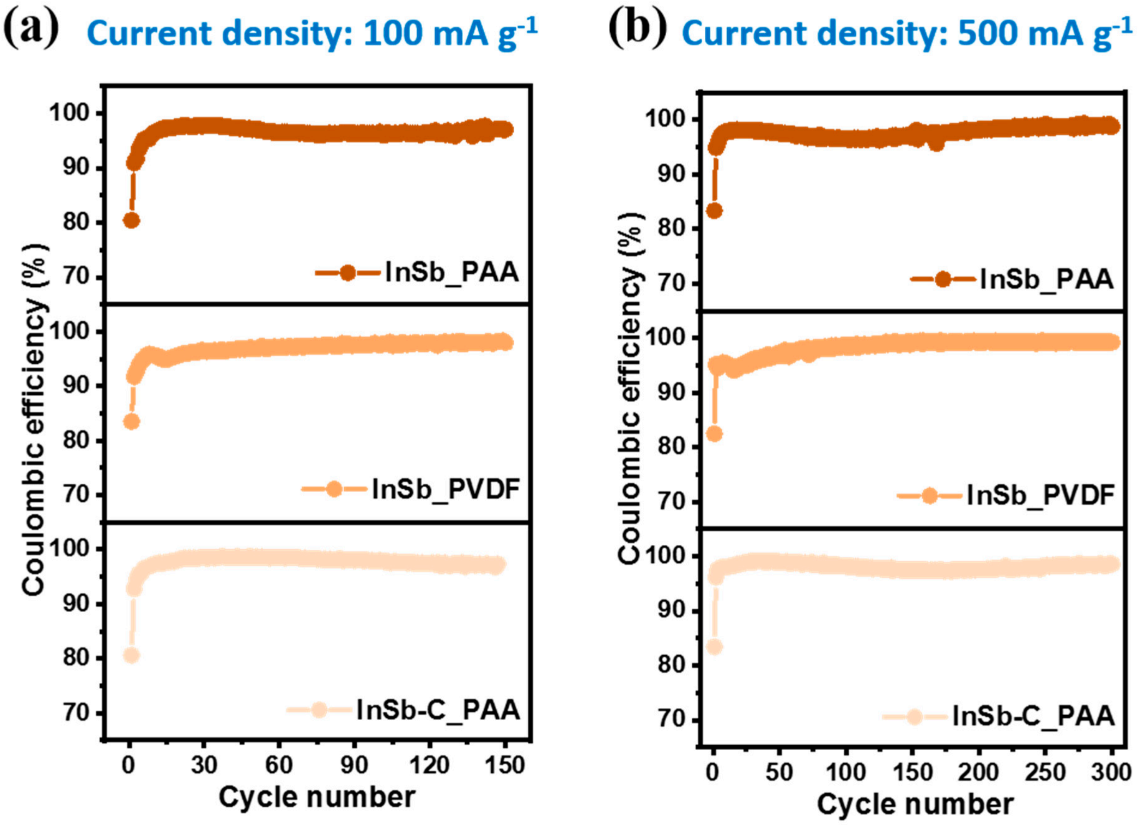

Figure S10. Coulombic efficieny of InSb_PAA, InSb_PVDF, and InSb–C_PAA at current density of (**a**) 100 and (**b**) 500 mA g^{-1}.

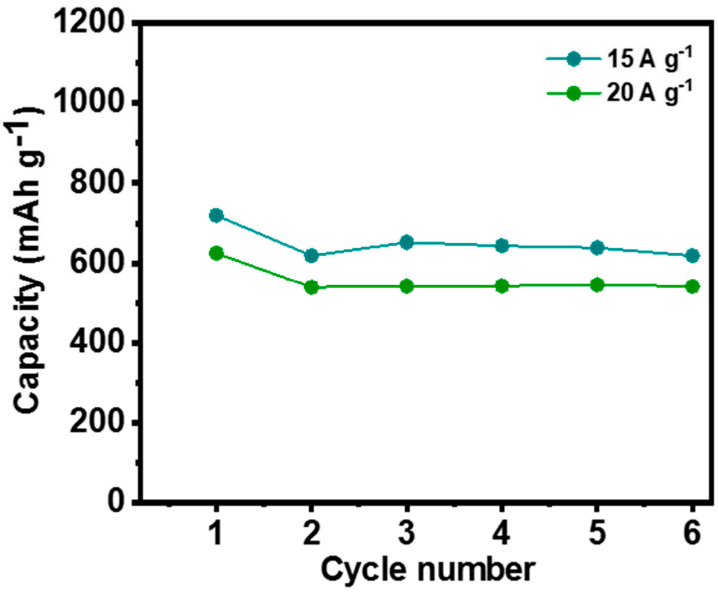

Figure S11. Cyclic performance of InSb-C_PAA at 15 A g^{-1} and 20 A g^{-1}.

Table S1. Crystallite size of InSb calculated using Scherrer equation.

	Facets	FWHM(β)/Radian	Crystallite size (nm)	Average size (nm)
InSb	(111)	0.0133	10.5504	5.3314
	(220)	0.0191	7.6469	
	(311)	0.0223	6.6994	
	(400)	0.0413	3.7726	
	(331)	0.0303	5.2875	
	(422)	0.0368	4.5826	
	(511)	0.0532	3.2761	
	(440)	0.2218	0.8355	

Scherrer formula: $= \frac{0.89\lambda}{\beta cos\theta}$, λ=0.1541 nm

Table S2. Coulombic efficiency variation of InSb_PAA at various cycle numbers measured at 100 mA g^{-1}.

Cycle number	Coulombic efficiency (%)
1	80.42
2	90.95
4	93.46
8	95.38
20	97.65
40	97.68
60	97.69
80	96.40
140	97.53

Table S3. Coulombic efficiency variation of InSb_PAA at various cycle numbers measured at 500 mA g^{-1}.

Cycle number	Coulombic efficiency (%)
1	83.35
2	94.88
4	95.99
8	97.61
20	97.63
40	97.80
60	97.39
80	97.25
100	96.53

Table S4. Calculation of capacity contribution of InSb and C in the InSb–C composite.

	**InSb**	**C**
The mole of Li-ion participating reaction	4	0.17
Molecular weight (g mol^{-1})	236.58	12
Calculated theoretical capacity (mAh g^{-1})	~454	~380
Actual weight faction in the InSb-C composite.	0.86	0.12
Contributed capacity (mAh g^{-1})	390	45.6
Capacity contribution (%)	90	10

Table S5. Calculation of theoretical capacity of InSb and InSb−C.

Anode material	InSb	InSb-C	
	InSb	InSb	C
Theoretical weight faction	0.98	0.86	0.12
Theoretical capacity (mAh g^{-1})	~454	~454	~380
Contributed theoretical capacity (mAh g^{-1})	~445	~390	~45.6
Total theoretical capacity (mAh g^{-1})	~454	~435.6	

Table S6. Coulombic efficiency of InSb_PAA, InSb_PVDF, and InSb–C_PAA at current density of 100 mA g^{-1} for initial 10 cycles.

**Cycle number**	**Coulombic efficiency (%)**		
	InSb_PAA	InSb_PVDF	InSb-C_PAA
1st	81.42	83.53	80.58
2nd	90.95	91.74	92.78
3rd	91.69	92.79	94.55
4th	93.46	93.89	95.55
5th	94.54	94.72	95.78
6th	95.31	95.13	96.57
7th	95.39	95.66	96.74
8th	95.37	95.84	96.90
9th	96.12	95.73	97.12
10th	96.48	95.68	97.32

Table S7. Coulombic efficiency of InSb_PAA, InSb_PVDF, and InSb–C_PAA at current density of 500 mA g^{-1} for initial 10 cycles.

**Cycle number**	**Coulombic efficiency (%)**		
	InSb_PAA	InSb_PVDF	InSb-C_PAA
1st	83.35	82.50	83.44
2nd	94.88	95.20	96.16
3rd	95.28	94.68	97.37
4th	95.99	94.63	97.62
5th	96.72	95.06	97.79
6th	97.18	95.25	97.97
7th	97.43	95.51	97.89
8th	97.61	95.35	98.02
9th	97.77	95.42	98.06
10th	97.83	95.28	98.06

Table S8. The charge-transfer resistance (R_{ct}) of InSb_PAA, InSb_PVDF, InSb–C_PAA.

	**InSb_PAA**	**InSb_PVDF**	**InSb–C_PAA**
1 cycle	54.29 Ω	48.44 Ω	116.74 Ω
5 cycles	38.17 Ω	105.91 Ω	51.92 Ω
20 cycles	21.56 Ω	55.68 Ω	19.37 Ω
